# Is There a Causal Relation between Maternal Acetaminophen Administration and ADHD?

**DOI:** 10.1371/journal.pone.0157380

**Published:** 2016-06-13

**Authors:** Antonio Saad, Shruti Hegde, Talar Kechichian, Phyllis Gamble, Mahbubur Rahman, Sonja J. Stutz, Noelle C. Anastasio, Wael Alshehri, Jun Lei, Susumu Mori, Bridget Kajs, Kathryn A. Cunningham, George Saade, Irina Burd, Maged Costantine

**Affiliations:** 1 Division of Maternal Fetal Medicine, Department of Obstetrics and Gynecology, University of Texas Medical Branch, Galveston, Texas, United States of America; 2 Integrated Research Center for Fetal Medicine, Division of Maternal Fetal Medicine, Department of Gynecology and Obstetrics, Johns Hopkins University, Baltimore, Maryland, United States of America; 3 The Russell H. Morgan Department of Radiology and Radiological Science, Johns Hopkins University, Baltimore, Maryland, United States of America; University of Rennes-1, FRANCE

## Abstract

**Objective:**

Recent epidemiological studies reported an association between maternal intake of acetaminophen (APAP) and attention deficit hyperactivity disorder (ADHD) in their children. However, none of these studies demonstrated causality. Our objective was to determine whether exposure to APAP during pregnancy result in hyperkinetic dysfunctions in offspring, using a murine model.

**Material and Methods:**

Pregnant CD1 mice (N = 8/group) were allocated to receive by gavage either APAP (150 mg/kg/day, equivalent to the FDA-approved maximum human clinical dose), or 0.5% carboxymethylcellulose (control group), starting on embryonic day 7 until delivery. Maternal serum APAP and alanine transaminase (ALT) concentrations were determined by ELISA and kinetic colorimetric assays, respectively. Open field locomotor activity (LMA) in the 30-day old mouse offspring was quantified using Photobeam Activity System. Mouse offspring were then sacrificed, whole brains processed for magnetic resonance imaging (MRI; 11.7 Tesla magnet) and for neuronal quantification using Nissl stain. The association between APAP exposure and LMA in mouse offspring was analyzed using a mixed effects Poisson regression model that accounted for mouse offspring weight, gender, random selection, and testing time and day. We corrected for multiple comparisons and considered P<0.008 as statistically significant.

**Results:**

Maternal serum APAP concentration peaked 30 minutes after gavage, reaching the expected mean of 117 μg/ml. Serum ALT concentrations were not different between groups. There were no significant differences in vertical (rearing), horizontal, or total locomotor activity between the two rodent offspring groups at the P level fixed to adjust for multiple testing. In addition, no differences were found in volumes of 29 brain areas of interest on MRI or in neuronal quantifications between the two groups.

**Conclusion:**

This study refutes that hypothesis that prenatal exposure to APAP causes hyperkinetic dysfunction in mouse offspring. Due to lack of accurate assessment of ADHD in murine models, our results should be taken with caution when compared to the reported clinical data.

## Introduction

Acetaminophen (APAP), marketed as Tylenol since the 1950s, is one of the most commonly used over the counter medications in the United States for pain and fever [1]. More than 50% of mothers in the United States report using acetaminophen during their pregnancy [2]. This is mainly due to APAP reported safety and favorable maternal-fetal safety profile. The exact mechanism of action of APAP is unknown.

APAP is known to cross the placenta, and recent studies reported that exposure to APAP during pregnancy may adversely influence fetal brain development by disrupting endocrine function and interfering with maternal hormones [2, 3]. Additionally, one study found an increased risk for hyperkinetic behavior in children who were exposed to APAP prenatally for more than one trimester, especially if the exposure was during the second and third trimesters. This risk was also more significant with increased duration and frequency of APAP use during pregnancy [2].

Attention deficit hyperactivity disorder (ADHD), a form of hyperkinetic dysfunction, is the most prevalent neurobehavioral disorder in children. In the U.S., there has been a rapid rise in ADHD diagnosis, and in 2011 approximately 11% of children (6.4 million) were diagnosed with ADHD [4]. ADHD is characterized by inattention, hyperactivity, impulsivity, and emotional dysregulation, [2, 5] and is usually diagnosed using select clinical criteria.

The reported association between APAP exposure and hyperkinetic dysfunction represents a dilemma for obstetricians since APAP is the most commonly used medication for pain or fever during pregnancy. In addition, the previously reported epidemiologic studies that reported the association between APAP exposure and hyperkinetic dysfunction did not address causality and were limited by their epidemiologic nature and potential for several confounders that may not have been accounted for. In view of these clinical and research dilemmas, we proposed to study whether exposure to APAP during pregnancy leads to hyperkinetic dysfunction in offspring, using a murine model.

## Materials and Methods

### Ethics Statement

This study was carried out in strict accordance with the recommendations in the Guide for the Care and Use of Laboratory Animals of the National Institutes of Health. All procedures involving animals were approved by the Institutional Animal Care and Use Committee (IACUC) at the University of Texas Medical Branch, Galveston, Texas (Protocol #0411077) and Johns Hopkins University, Baltimore, Maryland (Protocol #M011M420). All efforts were made to minimize suffering.

### Animals

Female CD-1 pregnant Swiss mice were purchased at embryonic day (ED) 5 from Charles River. Starting at ED 7 and until delivery (day 21), mice were randomly assigned to receive either APAP per gavage (150mg/kg/day) or 0.5% carboxymethylcellulose (control). This dose was chosen since it was previously shown to have the highest serum concentrations of acetaminophen without liver toxicity in mice [6–10, 11]. The observed mice serum concentrations at this dose were more than 10 fold higher than the measured human acetaminophen concentration after 1000 mg oral administration [6]. After weaning, dams were sacrificed and mouse offspring were placed in separate cages for locomotor testing. Timed pregnant CD-1 mouse dams (Charles Rivers Laboratories, Wilmington, MA) were individually housed in temperature- and humidity-controlled facility, with automatically controlled 12-hour light and dark cycles. Room temperature was kept at 26°Celsius which is within the thermoneutral range of our animal model. Certified personnel and veterinary staff provided regular maintenance and animal care according to IACUC guidelines. The general health status of the animals throughout the experiments, did not become ill or succumb prior to our experimental endpoints. The animals were sacrificed by CO2 inhalation per the American Veterinary Medical Association guidelines.

### Alanine Transferase (Alt) Colorimetric Assay

To assess liver injury, we measured dams’ serum ALT concentrations at baseline (ED 7) and at ED 18, using an ALT Activity Colorimetric Assay Kit (BioVision Inc., San Srancisco, CA). As per the ALT assay protocol, 20 μL of samples or standards was collected in each well along with 100μL of reaction mix (86 μL ALT assay buffer, 10 μL ALT substrate, 2 μL ALT enzyme mix, and 2 μL OxiRed probe). The absorbance was read at 570 nm after 10 minutes (A1) and then again after incubating the reaction at 37°C for 60 min (A2). Because the assay is run kinetically, the difference between A2 and A1 values needed to be obtained. A standard curve generated from this data was used to determine the concentration of pyruvate and activity of ALT in each sample.

### Acetaminophen Direct Elisa

On ED 16, serum APAP concentrations were measured before and then 30 and 60 minutes after APAP dosing, using Acetaminophen Direct ELISA (Immunalysis Corporation, Pomona, CA). Samples were diluted 1:2 with phosphate buffer saline. Each well contained 10μL of standards or samples and 100μL of enzyme conjugate. Samples were incubated at room temperature for 60 min. and washed. Substrate reagent (100 μL) followed by stop solution (100 μL) was added, taking note of the respective color change from blue to yellow. Absorbance was measured at 450 nm and corrected with 620 nm readings. The standard dose response curve generated from the data was used to determine the concentration of acetaminophen in each sample.

### Locomotor Testing

After weaning, 2 males and 2 females from each litter were randomly selected to undergo behavioral testing. Mice (postnatal day; PND 30) were placed in 16”x16” plexiglass enclosure for 2 hours/day over 3 days for habituation. Locomotor activity was quantified via Photobeam Activity System (PAS). To assess novelty induced hyperactivity and habituation, various locomotor parameters were measured by video tracking the animal throughout a session. The locomotor parameters included total locomotor activity (TLMA), rearing, time spent in the central versus peripheral part of the plexiglass enclosure, fine repetitive movements, and total ambulation [12].

### Brain Imaging

After locomotor testing, mouse offspring were sacrificed, whole brains processed for imaging and neuronal quantification. Magnetic resonance imaging (MRI) was done using 11.7 Tesla magnet and volumes of 29 brain areas were evaluated. These included nucleus accumbens, amygdala, anterior commissure, caudate/putamen, corpus callosum/external capsule, cerebellum, cingulum, claustrum, endoperiform, fimbria, fasciculus retroflexus, fornix, hippocampus, hypothalamus, inferior colliculus, internal capsule, lateral globus pallidus, neocortex, nosebud, olivary pretectal nucleus, periaqueductal gray, piriform cortex, septum, stria medullaris, submammillo-thalamic nucleus, stria terminalis, superior colliculis, thalamus, and ventricles. Both ex vivo and in vivo images were first rigidly aligned to a template image using automated image registration software (http://bishopw.loni.ucla.edu/AIR5/, AIR). After rigid alignment ex vivo images had the same position and orientation as the template image. Segmented images were submitted to Large Deformation Diffeomorphic Metric Mapping (LDDMM, www.mristudio.org), which assigns metric distances onto anatomical images and preserves topology and allows comparison of regional brain volume changes [13].

### Cytochemistry

Following MRI acquisition, whole brains were washed in 1X PBS for 30 min five times. The samples were processed for Nissl staining by immersing in 30% sucrose until saturation and cryosectioned at 20 μm thickness. All photographs used for quantification were taken with Zeiss AxioPlan 2 Microscope System (Jena, Germany) attached to a Canon EOS Rebel Camera (Tokyo, Japan) through a 40 objective lens. Neurons were counted (field of view) based on Nissl staining by using Image J (v1.48,http://imagej.nih.gov/ij/, National Institute of Health, Bethesda, MD) on randomly chosen 5 fields in frontal cortex per animal. The neurons were identified by a large cell body or perikaryon containing a large, pale nucleus with a prominent dark nucleolus. The experiments were performed in 3–5 repeats for all groups [14].

### Statistical Analysis

Analysis was performed using STATA (StataCorp, Dallas, Tx). Histology data, behavioral data, ALT and APAP concentrations are reported as mean ± S.E.M. for ALT and APAP comparisons, we used Student’s unpaired t-test unless otherwise stated, and a P value < 0.05 was considered statistically significant. For the locomotor activity evaluation, we used a mixed effects Poisson regression model that adjusted for mouse offspring weight, gender, random selection, and testing time and day. In order to correct for multiple comparisons, we considered a P value <0.008 to be statistically significant. The P value of 0.008 was chosen due to Bonferroni adjustment, since we are comparing simultaneously several dependent or independent variables on a single data set.

## Results

**Fig 1:**
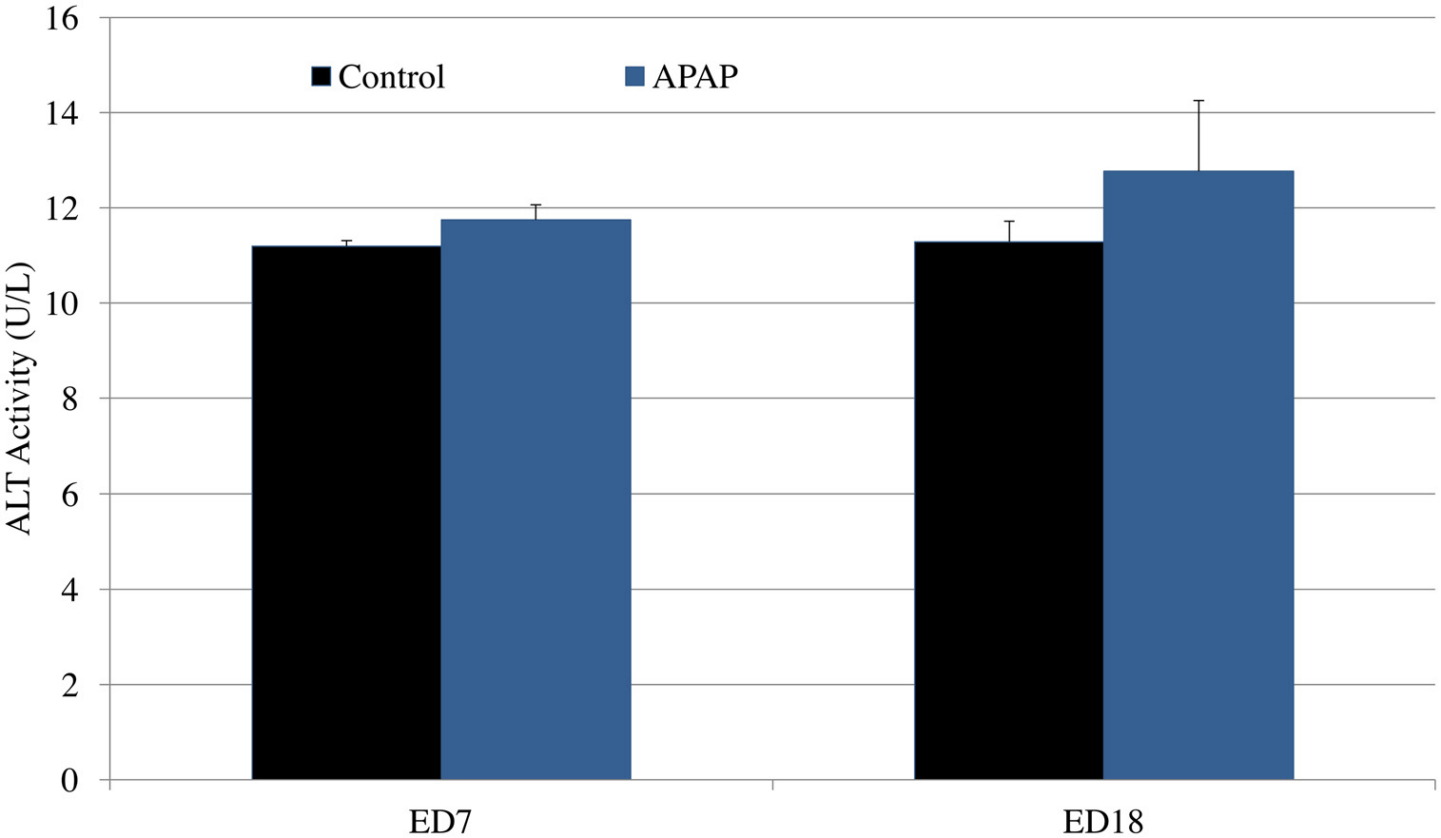
Serum ALT concentration in dams from control and APAP groups at ED 7 and 18.

Maternal serum concentration of APAP peaked at 30 min after gavage, reaching a mean of 117 μg/ml. This concentration is in range with what was previously reported in APAP pharmacokinetic studies in human and mice [6–11]. APAP concentrations in the control group were below the lowest level of quantification of the assay (10 μg/ml). At baseline (ED 7) and then at ED 18, there were no differences in maternal serum ALT concentrations between the two groups of the study (Fig 1, 11.2 vs 11.7, P = 0.3;11.7 vs 12.7, p = 0.5).

**Fig 2:**
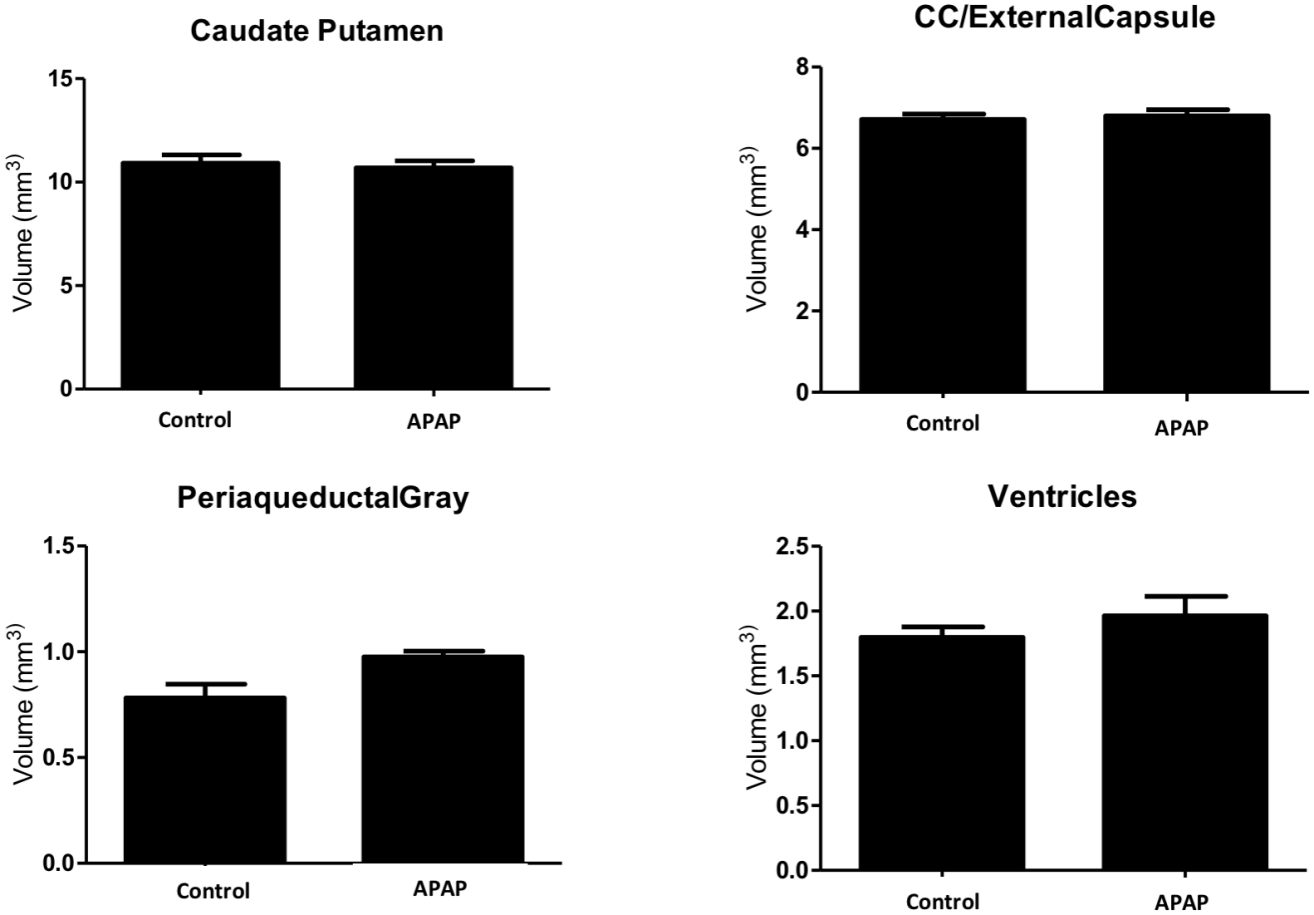
Computer-generated brain volumes of offspring mice as determined by MRI. MRI was done using 11.7 Tesla magnet and volumes of 29 brain areas were evaluated. No differences were found in volumes of brain locations of interest on MRI. Representative areas relevant to ADHD are illustrated. Data are presented as the mean ± s.e.m. Statistical significance was determined using Student’s unpaired t-test.

**Fig 3:**
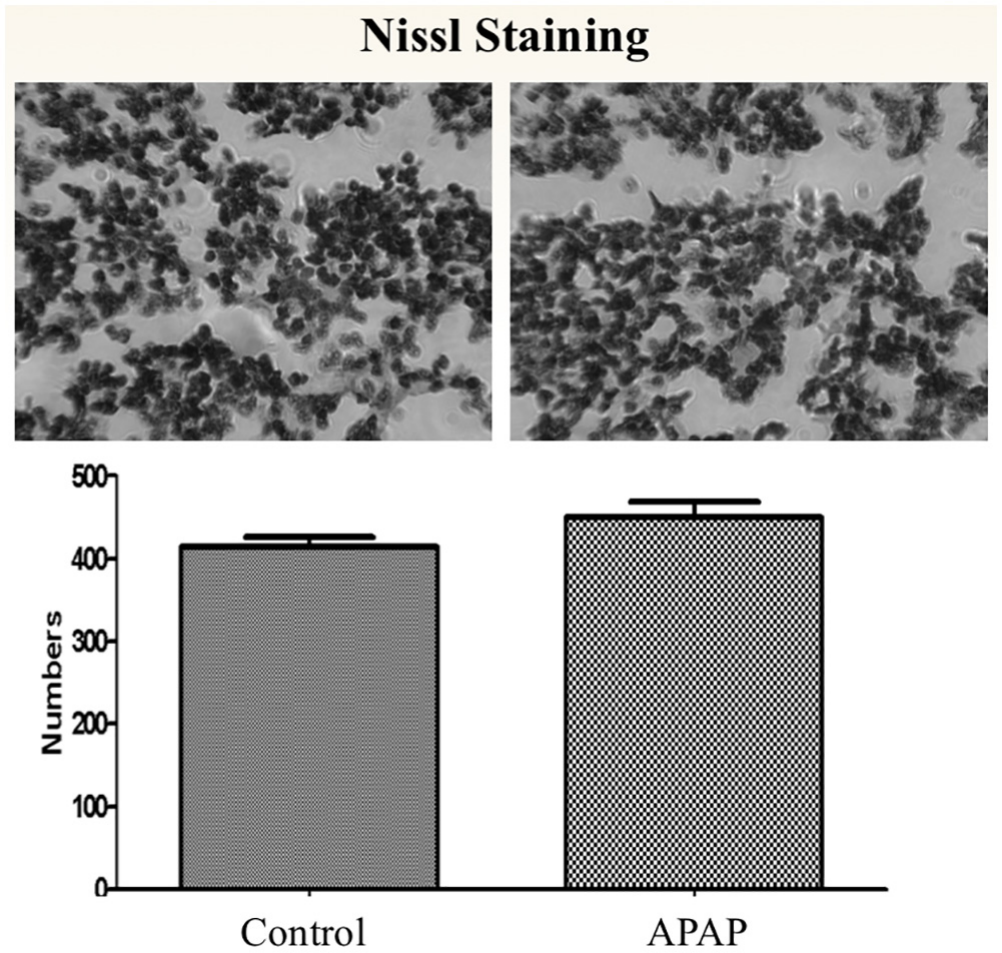
Cortical neuron densities in Nissl-stained frontal cortex of offspring between control and APAP groups. Whole brains were washed and processed for Nissl staining by immersing in 30% sucrose until saturation and cryosectioned at 20 μm thickness. Neurons were counted (field of view) based on randomly chosen 5 fields in frontal cortex per animal. The neurons were identified by a large cell body or perikaryon containing a large, pale nucleus with a prominent dark nucleolus. Experiments were performed in 3–5 repeats for all groups. There was no significant difference in cortical neuron density between control and treated groups (p < 0.05; Unpaired Student’s T-Test; n = 5 control, n = 3 treated). Data are presented as the mean±s.e.m. Statistical significance was determined using Student’s unpaired t-test.

There were no significant differences in any locomotor activity parameter between the two groups of rodent offspring at the P level fixed to adjust for multiple testing (Table 1). The parameters observed were total locomotor activity (TLMA), vertical activity (rearing), spending more time in the central versus peripheral areas of the enclosure, fine repetitive movements, and total ambulation. In addition, when we accounted for gender alone in the analysis, the results did not differ among males or females mouse offspring (Table 1). Table 2 summarizes the observed original mean values for each observed parameters. Moreover, we did not find any significant differences in volumes of 29 brain areas of interest between the two groups (Fig 2) or in neuronal quantification of Nissl-stained cryo-sections of the frontal cortex (Fig 3). Normal brain architecture was noted in both groups, with no evidence of disarray or apoptosis in the cortical neurons. Representative coronal and axial brain areas are illustrated in S1 Fig.

**Table 1:** Incidence rate ratio (95% confidence interval) of different activities for treatment group compared to control group: mixed effects poisson regression analysis that adjusted for offspring weight, gender, random selection, and testing time and day. P<0.008 was considered significant.

		TLMA	Rear	Central	Peripheral	Fine	Total Ambulation
Treatment group (vs. control group)	Overall Unadjusted IRR (95%CI) *P* value	1.115 (0.951–1.308) *P* = .180	1.123 (0.903–1.396) *P* = .296	0.996 (0.788–1.258) *P* = .971	1.132 (0.962–1.333) *P* = .136	1.092 (0.945–1.262 *P* = .231	1.134 (0.953–1.350) *P* = .157
	Overall Adjusted IRR (95%CI) *P* value	1.140 (0.989–1.315) *P* = .071	1.148 (0.939–1.403) *P* = .179	0.976 (0.777–1.225) *P* = .832	1.167 (1.007–1.352) *P* = .040	1.113 (0.979–1.266) *P* = .102	1.181 (1.007–1.385) *P* = .041
	Adjusted IRR for Male Offspring (95% CI) *P* value	1.121 (0.934–1.569) *P* = .147	1.325 (1.012–1.737) *P* = .041	0.936 (0.685–1.279) *P* = .680	1.257 (0.980–1.611) *P* = .071	1.220 (1.020–1.460) *P* = .029	1.211 (0.978–1.499) *P* = .079
	Adjusted IRR for Female Offspring (95% CI) *P* value	0.986 (0.797–1.219) *P* = 0.893	0.932 (0.697–1.246) *P* = .635	0.951 (0.734–1.233) *P* = .705	0.978 (0.782–1.234) *P* = .846	0.926 (0.777–1.102) *P* = .387	1.039 (0.818–1.320) *P* = .752

*TLMA*, total locomotor activity was calculated from addition of central and periphery gross motor activity and fine activity. N = 16/group.

**Table 2:** Mean values of different activities according to group assignment.

GROUP ASSIGNMENT	TLMA	Rear	Central	Peripheral	Fine	Total Ambulation
CONTROL	106.4 ± 2.5	27.6 ± 0.7	13.7 ± 0.4	92.6 ± 2.2	41.1 ± 0.8	65.3 ± 1.8
ACETAMINOPHEN	115.8 ± 2.8	29.5 ± 0.7	14.2 ± 0.4	101.6 ± 2.5	44.5 ± 0.9	71.3 ± 1.9

*TLMA*, total locomotor activity was calculated from addition of central and periphery gross motor activity and fine activity. N = 16/group. Data are presented as the mean ± s.e.m. Unis are number of events.

Pertinent data of behavioral, ALT and APAP assays are found in S1 File.

## Discussion

Two observational studies reported an association between maternal APAP exposure and offspring development of ADHD-like behaviors. The first [2] reported a small increased risk for hyperkinetic behavior in children, that correlated with increased duration and frequency of maternal APAP usage [2]. A more recent study [15] supported the prior association and showed an association between APAP and ADHD symptoms at age 7 and 11. The increased risk remained after adjusting for potential confounders such as fever, stress, alcohol, smoking and inflammatory problems [15].

Despite the above epidemiological evidence, the results of the two studies should be taken with caution. The current evidence from the observational epidemiological studies supports a mere association and not causation. The epidemiological nature of these studies makes the results limited by unmeasured confounders such as differences in pain tolerance and indications to use the drug in the first place. These and other confounders may be more associated with ADHD-like behavior compared with ingestion of the drug itself. In addition, these associations were found with long term (weeks and months) APAP administration which does not typically occur when treating pregnant women. The limitations of the observational studies led professional societies such as the American College of Obstetricians and Gynecologists and the Society of Maternal Fetal Medicine to release statements supporting the use of acetaminophen in pregnancy, as it is still considered one of the safest medications in pregnancy with minimal fetal risks when compared to other anti-inflammatory drugs such as NSAIDS which can lead to fetal kidney failure or narcotic medications. As a general rule, observational studies do not demonstrate causality, and this led us to conduct our study to demonstrated or refute a causal association.

Our objective was to elucidate the causal relationship between prenatal APAP exposure and hyperactive phenotype characteristic of ADHD in a murine model. To our knowledge, this is the first study assessing causality between maternal exposure to APAP and risk of ADHD in mouse offspring. Findings from this study do not support a causal relationship.

In this study, pregnant mice were administered APAP at a dose of 150 mg/kg/day. Administration of this dosage in mice did not result in any hepatotoxicity as measured by serum ALT activity. While we were able to appropriately administer the upper limit of a normal dose, without liver damage, our behavioral studies indicate no differences in locomotor activity between the two groups. In addition, we started APAP at ED 7 and continued until delivery. This corresponds to APAP administration throughout the second and third trimesters. While this is not the typical regimen that most pregnant women will take, it supports our original hypothesis that the prior epidemiologic studies were most likely confounded by various variables that possibly were not accounted for, since a maximal dose for the latter half of pregnancy did not reproduce the phenotype.

Behavioral testing was conducted on the mouse offspring at post-natal day 30, the human equivalent of childhood/adolescence [16], the time period during which ADHD symptoms are more likely to manifest. Mice displaying ADHD-like behavior were expected to have greater total locomotor activity, ambulation, and fine repetitive movements such as scratching, washing, chewing, etc. Since ADHD is closely intertwined to anxiety disorders, we expected mice with ADHD phenotype to spend less time in the periphery (suggestive of exploratory behavior) and more time centrally (suggestive of anxious behavior) in open arena sessions. Rearing behavior, a vertical activity in which the mice stand on their hind legs, was also more suggestive of exploratory behavior and not expected in those displaying ADHD-like behavior [17]. Our findings of no significant differences in locomotor activity between the two groups, indicate that APAP administration did not result in any remarkable hyperkinetic dysfunction as compared to the control group.

Moreover, various neuroimaging studies of patients with ADHD showed deviations in asymmetric caudate-striatal morphology, [18] and decreased volumes of the cerebrum, cerebellum, and basal ganglia region, particularly globus pallidus and striatum [18]. In our study we performed MRI analysis of more than 29 brain areas, and did not observe any significant differences in brain volumes or morphology between the groups, including caudate-striatal areas relevant to ADHD, supporting our hypothesis that even APAP administration prenatally is not associated with the phenotype or candidate brain areas associated with ADHD pathophysiology.

In addition, neuronal disarray and increased pyknotic and apoptotic activity is thought to be associated with pathophysiology of neurodevelopmental disorders such as ADHD [19]. In our study, further histologic examination of the prefrontal cortex in the mouse offspring using Nissl-staining, showed no differences in arrangement, size, or structure of the cortical densities; further supporting our findings that APAP exposure does not seem to cause any behavioral, anatomical, or histological abnormalities in mice offspring brain.

One of the limitations of our study is that since there is no established animal model for ADHD, we could not have a positive control to which the APAP treated group could be effectively compared. In addition, the optimal control group should have been dams not receiving treatment, since oral gavage itself may act as a stressor and could have masked our lack of differences between groups. Less stressful drug delivery methods are needed for future research.

Because brain development continues in the postnatal period, APAP should have been administered during the lactation period. However, due to concerns that nurturing disruption itself may affect the pup’s behavior pattern, thus confounding our results; we opted to terminate interventions after birth. Moreover, since the goal of this study was to determine whether exposure to APAP during pregnancy result in hyperkinetic dysfunctions in mouse offspring, we administered the drug during pregnancy alone.

The study was also limited by the fact that we did not perform 24hr home cage activity, which may better fit the diagnostic criteria of ADHD and ADHD-like hyperactivity. In addition, we chose to observe for differences in hyperkinetic behavior between the two groups. This narrowed focus may be a limitation since we did not investigate other variables that comprise the ADHD spectrum disorder including inattention, impulsivity, and emotional dysregulation. However, the hyperkinetic dysfunction is a major component of ADHD, and the prior epidemiologic studies suggested that APAP is associated with this component. Due to lack of accurate assessment of ADHD in murine models, our results should be taken with caution when compared to the reported clinical data. Future studies should include examining other ADHD characteristics and should focus in developing better ADHD animal models.

In conclusion, the etiology of ADHD remains unknown and complex, likely resulting from interaction of genetic, epigenetic, and environmental factors [20]. This is the first study assessing the role of APAP prenatal exposure and risk of ADHD, using a murine model. Our results do not support a causal relationship. Results of epidemiological studies may be due to confounding factors that were not accounted for. Since performing a prospective study in human patients may be difficult and possibly impossible due to costs and obvious ethical reasons, further animal studies are required to assess other ADHD phenotypes not assessed in the current manuscript.

## Supporting Information

S1 Fig Brain Volumes in postnatal day 30 offspring. Three-dimensional T2-weighted images of whole brain were obtained using a vertical bore 11.7 Tesla MRI scanner. Coronal and axial representative areas are illustrated. CC, Corpus Callosum. R, right; L, left.
(TIFF)

S1 File Raw data for the behavioral, ALS and APAP assays. (ZIP)
