# Supplementary figures and images for: Is There a Causal Relation between Maternal Acetaminophen Administration and ADHD?
Source: PLoS One. 2016 Jun 13;11(6):e0157380. doi: 10.1371/journal.pone.0157380 (PMC4905664; doi:10.1371/journal.pone.0157380)

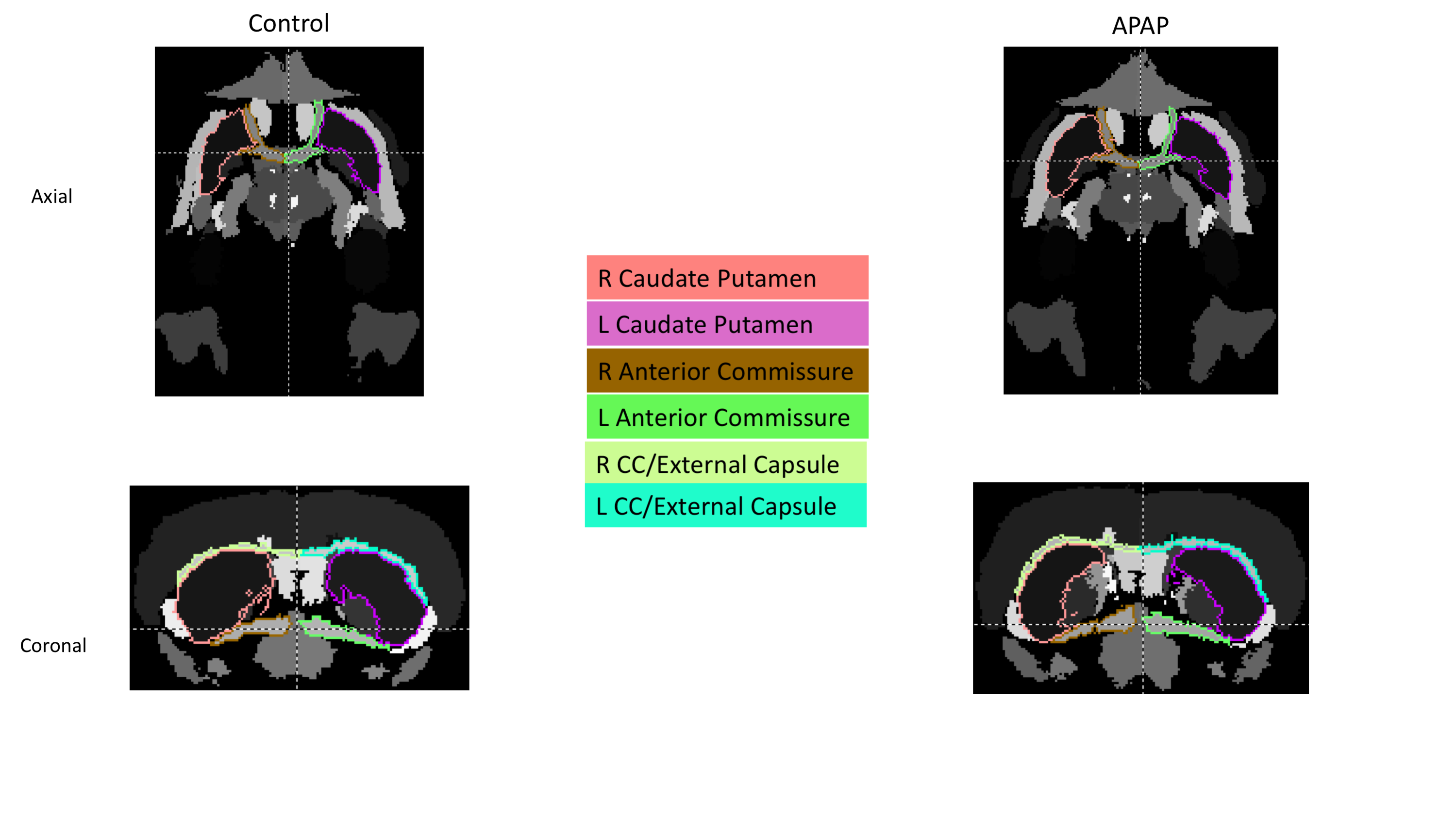

Supplement: S1 Fig — Three-dimensional T2-weighted images of whole brain were obtained using a vertical bore 11.7 Tesla MRI scanner. Coronal and axial representative areas are illustrated. CC, Corpus Callosum. R, right; L, left. (TIFF) [file pone.0157380.s001.tiff]
